# Psyllium seed husk regulates the gut microbiota and improves mucosal barrier injury in the colon to attenuate renal injury in 5/6 nephrectomy rats

**DOI:** 10.1080/0886022X.2023.2197076

**Published:** 2023-04-05

**Authors:** Dongmei Hu, Wenbo Liu, Wanlin Yu, Lihua Huang, Chunlan Ji, Xusheng Liu, Zhaoyu Lu

**Affiliations:** a State Key Laboratory of Dampness Syndrome of Chinese Medicine, The Second Clinical College of Guangzhou University of Chinese Medicine, Guangzhou, China; b Nephrology Department, The Second Affiliated Hospital of Guangzhou University of Chinese Medicine, Guangzhou, China

**Keywords:** Chronic kidney disease, psyllium seed husk, gut microbiota, colon mucosal barrier, renal-protective

## Abstract

Chronic kidney disease (CKD) can cause gut microbiota dysbiosis and thus impair intestinal barrier function. Disruption of intestinal homeostasis facilitates the production of enterogenic toxins, which exacerbate CKD-induced uremic toxicity and inflammation. Dietary fiber, by targeting the gut–kidney axis, could be used for CKD treatment. Psyllium seed husk (PSH) extracted from the seeds of *Plantago ovata* contains highly branched, gel-forming arabinoxylan. Positive effects of PSH on host physiology have been demonstrated but whether it also acts on the microbial ecosystem in CKD patients is unknown. In this study, the effects of dietary PSH on the gut microbiota, intestinal barrier function, systemic inflammation, uremic toxins, and renal injury were investigated in 5/6 nephrectomy (5/6Nx) CKD rats. Blood, feces, and kidney and colon tissues were collected from PSH-treated and control rats and subjected to biochemical and histological analyses, enzyme-linked immunosorbent assays, and 16SrRNA sequencing. PSH supplementation reduced serum creatinine and blood urea nitrogen levels, and attenuated renal tubular interstitial injury, in 5/6Nx rats. 16SrRNA sequencing showed that PSH improved the gut microbiota and intestinal barrier function in addition to down-regulating serum interleukin (IL)-1, IL-6, and indoxyl sulfate levels. Together, these results demonstrate the potential of PSH supplementation for treating CKD, including by improving intestinal microecology, reducing uremic toxin levels and systemic inflammation, and delaying disease progression.

## 1. Introduction

Chronic kidney disease (CKD) is a global public health problem and its incidence has been increasing annually. Most patients with CKD will develop end-stage renal disease, which has a poor prognosis and entails high healthcare costs [1].

Several studies [2,3] have demonstrated a bidirectional interaction of the kidney and gut in CKD, referred to as the gut–kidney axis. CKD can lead to an imbalance of the gut microbiota and impaired intestinal barrier function [4], with the destruction of intestinal homeostasis promoting the production of uremic toxins. The translocation of intestinal bacteria and endotoxins results in systemic inflammatory and oxidative stress, renal fibrosis, and renal tubular damage, thereby accelerating CKD progression [5,6].

Dietary fiber consists of carbohydrate polymers that are neither digested nor absorbed in the upper digestive tract; they enter the colon directly, where they are partially or completely fermented by colonic bacteria. It includes both natural plant foods and health-promoting compounds isolated from natural and artificially synthesized materials [7]. Among the positive effects of dietary fiber on human health are improved integrity of the gastrointestinal wall and a reduction in the levels of systemic uremic toxins [8]. Accordingly, dietary fiber may be even more important for patients with CKD. In the National Health and Nutrition Examination Survey III, which enrolled 14,543 participants, increased dietary fiber intake was associated with statistically and clinically significant reductions in inflammation and mortality due to kidney disease. The effect was significantly more pronounced in participants with than without CKD [9]. Despite these findings, nutritional guidelines for patients with kidney disease [10] make weak recommendations regarding dietary fiber, as there is insufficient evidence from clinical and laboratory studies. Therefore, further studies on the role of dietary fiber in CKD are clearly warranted.

Psyllium seed husk (PSH) is a water-soluble, gelatinous mucilage extracted from *Plantago ovata* (blond plantain) [11]. PSH supplementation can effectively reduce the risk of cardiovascular diseases [12], improve blood lipid profiles, and regulate gut microbiota [13]. In a recent trial, plantain supplementation reduced the inflammatory response to colitis in mice. While this response was found to be largely dependent on the microbiome [14], the effects of PSH on the microbial ecosystem of CKD have not yet been investigated in detail. Therefore, in this study, the effects of PSH, as a representative dietary fiber, on the gut–kidney axis were examined in a well-described 5/6 nephrectomy (5/6Nx) rat model of CKD.

## 2. Materials and methods

### 2.1. Experimental animals

Eight-week-old male Sprague-Dawley rats were purchased from the Animal Center of Southern Medical University (Guangzhou, China). Prior to the start of the experiment, the animals were housed in specific pathogen-free animal breeding rooms in a temperature-controlled environment with a 12-h light–dark cycle, and allowed free access to food and water. The experiments were conducted in accordance with protocols for laboratory animal care and use approved by the Institutional Ethics Review Board of Guangdong Provincial Hospital of Chinese Medicine.

### 2.2. Surgical procedures for 5/6 nephrectomy rats and PSH administration

To induce CKD, bilateral kidneys of the rats were excised 5/6Nx. During the operation, the rats were anesthetized with 2% pentobarbital sodium intraperitoneally. The left kidney was externalized, two-thirds was removed and hemostasis was achieved using 3M tissue adhesive. One week later, the rats were anesthetized, the right kidney was externalized on the back, the renal pedicle was sutured with 4-0 silk suture, the right kidney was removed, and a 5/6Nx model was performed. As for the sham-operation group rats, the kidney was only isolated and then collected without Nx.

Serum creatinine (SCr) and blood urea nitrogen (BUN) levels were measured 4 weeks later. The rats were then randomly divided into four groups (*n* = 10 each): sham; untreated 5/6Nx + placebo (model); 5/6Nx treated with PSH-low dose (psyllium L, 250 mg/kg/day); and 5/6Nx treated with high-dose PSH (Psyllium H, 500 mg/kg/day). In the PSH-treated groups, the rats were fed PSH for 30 days. PSH containing 25% dietary fiber was provided by Now Foods Company (Bloomingdale, IL).

### 2.3. Biochemical detection

Serum creatinine and BUN were measured using a Roche Cobas C702 automatic analyzers (Tokyo, Japan). Proteinuria was detected using a BCA protein assay kit (Thermo Fisher Scientific, Waltham, MA).

### 2.4. Histopathological studies

Paraffin-embedded rat kidney and colon tissues were cut into 3-µm sections. Kidney sections were stained with hematoxylin and eosin (H&E), periodic acid-Schiff (PAS), and Masson’s trichrome. Colon sections were stained with H&E. Kidney fibrosis was measured quantitatively using ImageJ (NIH, Bethesda, MD). Tubulointerstitial injury was assessed semi-quantitatively according to a previously reported method [15]. The lesions were graded from 0 to 3 (0, no changes; 1, changes affecting <25% of the section; 2, changes affecting 25–50% of the section; 3, changes affecting 51–100% of the section) according to the area occupied by tubulointerstitial injury (tubular atrophy, casts, interstitial inflammation, and fibrosis).

### 2.5. Enzyme-linked immunosorbent assay

Serum levels of interleukin (IL)-1β, IL-6, indoxyl sulfate (IS), and diamine oxidase (DAO) were determined by enzyme-linked immunosorbent assay (ELISA) performed according to the manufacturer’s instructions. Rat IL-1β and IL-6 high-sensitivity ELISA kits were purchased from Absin (Shanghai, China). The IS assay kit was obtained from Creative Diagnostics (Shirley, NY), and the DAO ELISA kit was from Cusabio (Wuhan, China).

### 2.6. Immunohistochemistry

Paraffin-embedded colon sections were dewaxed, rehydrated, and immersed in 3% hydrogen peroxide for 10 min at room temperature to block endogenous peroxidase activity, followed by antigen retrieval for 15 min. All sections were blocked for 30 min with 5% blocking buffer. The sections were incubated with anti-occludin primary antibody (1:100, lot#:GR3225940-2; Abcam, Cambridge, UK), anti-claudin-1 primary antibody (1:100, lot#:GR3285466-5; Abcam, Cambridge, UK) at 4 °C overnight. The next day, the sections were washed and then incubated with species-specific secondary antibody (Boster, Wuhan, China), developed with 3,3′-diaminobenzine (Boster, Wuhan, China), and counterstained with hematoxylin. The integrated optical density of positively staining areas was measured using ImagePro Plus 6.0 software (Media Cybernetics, Silver Spring, MD).

### 2.7. Processing of 16SrRNA gene sequences

Stool DNA was extracted using a NucleoSpin soil kit (Macherey-Nagel, Biocompare, Düren, Germany). PCR was performed using 30 ng of the genomic DNA sample and the corresponding primers. The PCR-amplified products were purified, dissolved in elution buffer using Agencourt AMPure XP magnetic beads, and labeled to complete library construction. Sequencing was carried out on the Illumina Hiseq 2500 platform (Illumina, San Diego, CA) based on the size of the fragments inserted.

### 2.8. Bioinformatics and biostatistics

Raw sequencing data were filtered to obtain clean reads by eliminating adapter contamination and low-quality reads. Overlapping reads were merged into labels using FLASH (version 1.2.11) and then clustered into operational taxonomic units (OTUs) using USEARCH (version 7.0.1090) based on 97% sequence similarity. A ribosome database item classifier (version 2.2) trained on the Greengene_2013_5_99 database was used to assign taxonomic grades to representative OTUs, with a confidence value of 0.6 used as the cut-off.

All statistical analyses were performed using R software (version 3.5.0; R Foundation for Statistical Computing, Vienna, Austria). α-diversity (Shannon and Chao indices) determination and partial least squares discrimination analysis (PLS-DA) were performed at the genus level. 16SrRNA gene sequences were analyzed using the *vegan* package, which was also used to perform permutation multivariate ANOVA of dissimilarity matrices to assess the effect of 10,000 permutations. A linear discriminant analysis of effect size (LEfSe) was used to analyze group characteristics and the effect sizes of significant differences between groups. EnvFit analysis was used to determine the effect sizes and significance of the covariates in each group of 999 permuted plasmids. A redundancy analysis (RDA) was performed using the *vegan* package. Correlations between genera and covariates, adjusted for groups, were identified by general linear modeling. The false discovery rate was used to assess the significance of differences, with *q* < 0.1. Based on the MetaCyc database, pathoLogic (part of the Pathway Tools software) was used to predict the metabolic network. MetaFlux (Pathway Tools) was used to convert those tools into a metabolic model, which was then used to compare the metabolic pathways of different groups.

### 2.9. Statistical analyses

SPSS software (version 18.0; SPSS Inc., Chicago, IL) was used for all statistical analyses. The results are expressed as mean ± standard deviation. ANOVA was used for the group comparisons. A *p* value <.05 was considered to indicate statistical significance (*) and a *p* value <.01 indicated highly statistically significant results (**).

## 3. Results

### 3.1. PSH improves biochemical parameters in 5/6Nx rats

**Figure 1.**
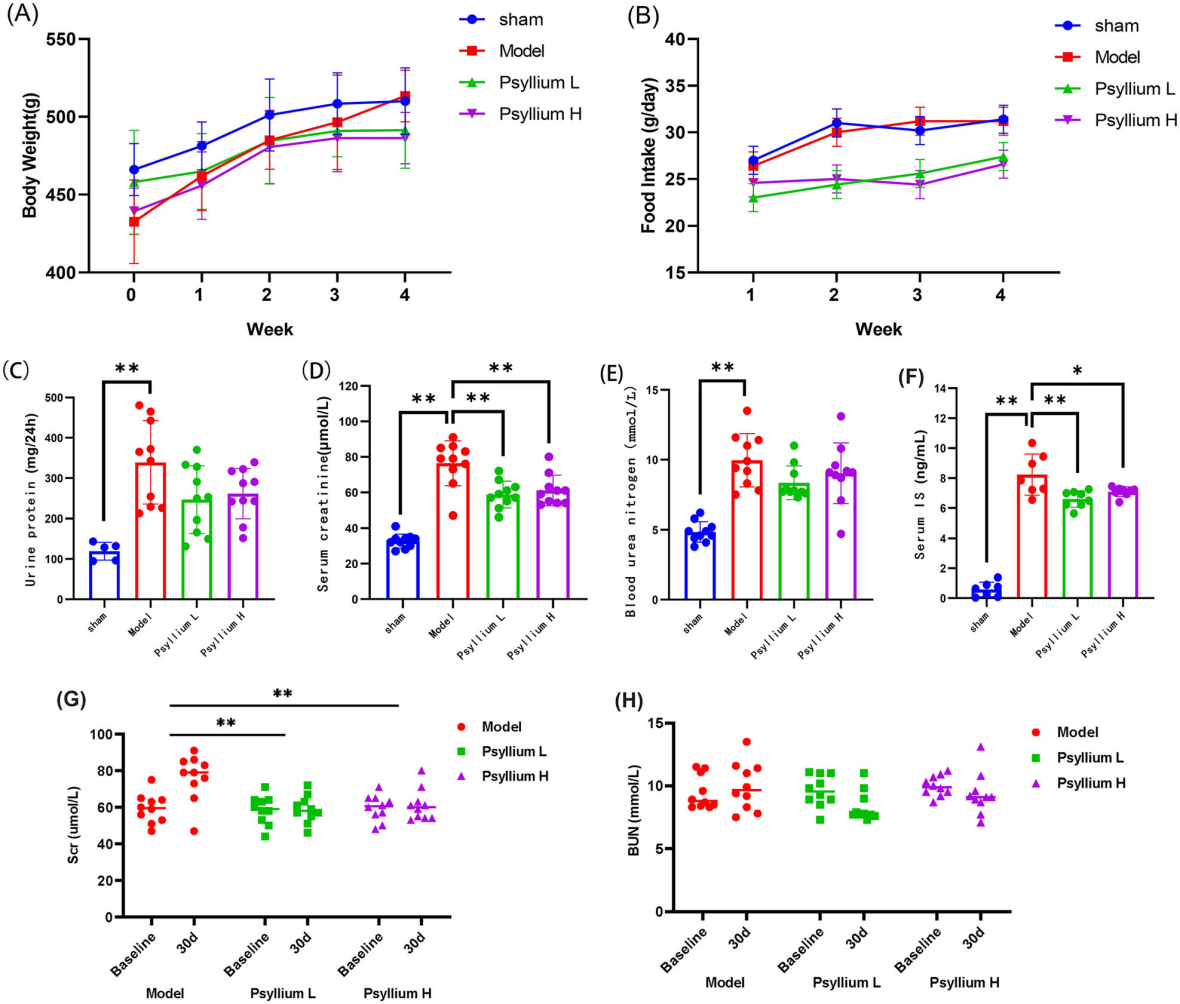
Effects of psyllium seed husk (PSH) on 5/6 nephrectomy (5/6Nx) rats. (A) Body weight; (B) food intake; (C) 24-h urine protein; (D) serum creatinine (SCr); (E) blood urea nitrogen (BUN); (F) serum level of indoxyl sulfate (IS). (G) ΔSCr from baseline to the end point. (H) ΔBUN from baseline to the end point. Data are presented as mean ± standard deviation for each measurement (*p<.05, **p<.01).

Neither body weight nor food intake differed significantly between the sham and 5/6Nx groups. However, food intake was reduced in Psyllium L and Psyllium H rats, resulting in a trend toward a lower body weight than in 5/6Nx rats (Figure 1(A,B)).

Significant proteinuria developed in 5/6 Nx rats, but proteinuria levels were lower in the Psyllium L and Psyllium H rats (Figure 1(C)). Both SCr and BUN levels were significantly higher in 5/6Nx than sham rats (*p*<.01, Figure 1(D,E)). SCr and ΔSCr were significantly lower (*p*<.01, Figure 1(D,G)), and BUN and ΔBUN slightly lower, in PSH-treated 5/6Nx rats than in untreated (model) 5/6Nx rats (Figure 1(E,H)). There was no difference between Psyllium L and Psyllium H rats (Figure 1(D,E,G,H)).

### 3.2. PSH supplementation reduces kidney damage in 5/6Nx rats

**Figure 2.**
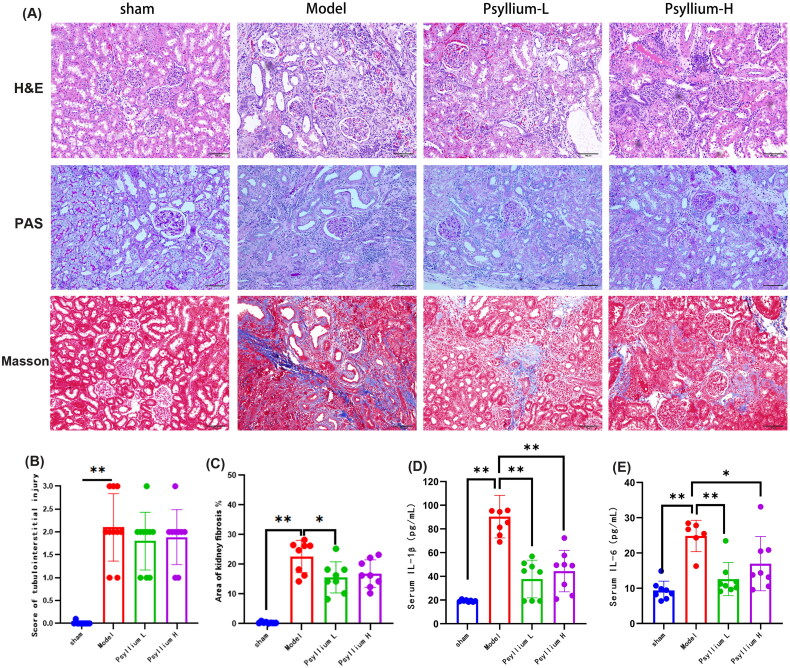
PSH inhibits kidney damage and systemic inflammation in 5/6Nx rats. (A) Hematoxylin and eosin (H&E), periodic acid-Schiff, and Masson staining of kidney tissue (×100); (B) tubulointerstitial injury; (C) area of kidney fibrosis; (D) serum levels of interleukin (IL)-1β; (E) serum levels of IL-6. Data are presented as mean ± standard deviation (*p<.05, **p<.01).

To determine whether the therapeutic effect of PSH was mediated by reductions in SCr and BUN levels, renal pathology was examined by H&E, PAS, and Masson staining of kidney sections. In untreated 5/6Nx rats, renal tubule lumen dilation and atrophy, mononuclear lymphocyte infiltration, and interstitial fibrosis were observed (Figure 2(A)), whereas in the sham group there was no evidence of tubule or tubulointerstitial injury. Both the tubulointerstitial injury score and area of kidney fibrosis were significantly greater in the model than sham group (Figure 2(B,C)). Renal injury in 5/6Nx rats improved significantly after Psyllium L treatment, but there was no significant difference between the Psyllium L and Psyllium H treatments (Figure 2(A–C)). These results indicated that PSH supplementation slows the progression of renal injury in 5/6Nx rats.

### 3.3. PSH supplementation reduces inflammation and uremic toxins

The relationship between renal injury and regulation of the gut–kidney axis was assessed by first measuring serum levels of the inflammatory cytokines IL-1β and IL-6, and the uremic toxin IS.

Serum concentrations of IL-1β, IL-6, and IS were significantly higher in model 5/6Nx than sham rats (*p*<.01) (Figures 1(F) and 2(D,E), respectively). Both low- and high-dose PSH treatment significantly reduced IL-1β, IL-6, and IS levels in 5/6Nx rats (**p<.01, *p<.05, Figure 2(C–E)), but there was no significant difference between the low- and high-dose PSH groups.

### 3.4. PSH supplementation changes the gut microbiota

Since high-dose PSH was not superior to low-dose PSH, gut microbiota analysis using 16SrRNA gene sequencing was conducted only in the sham, 5/6Nx, and Psyllium L groups.

**Figure 3.**
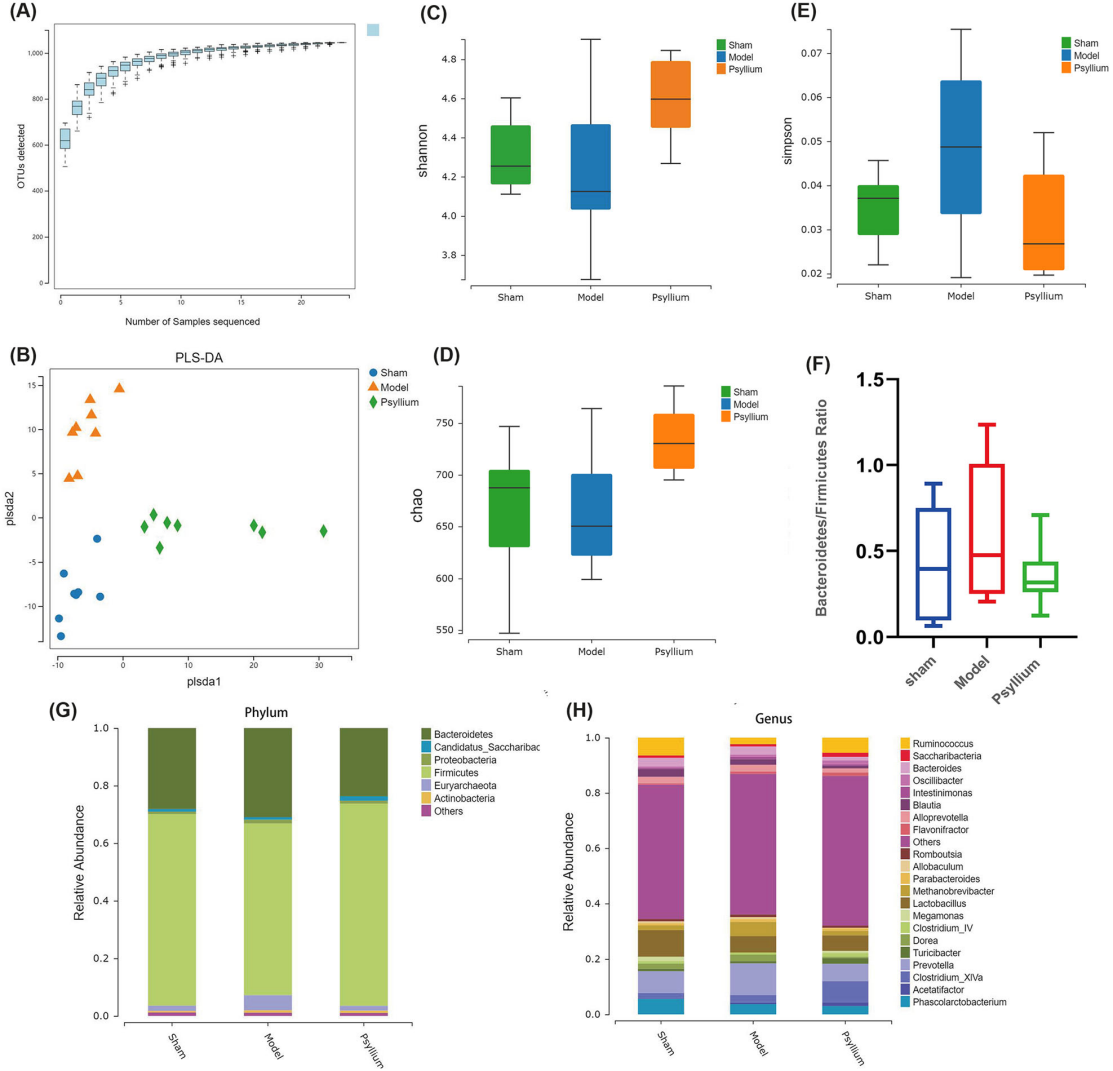
Regulation of PSH of the gut microbiome in 5/6Nx rats. (A) Species accumulation curve; (B) partial least squares discrimination analysis comparing the β-diversity of gut microbiota; (C) Shannon index (α-diversity); (D) Chao index (α-diversity); (E) Simpson index (α-diversity); (F) Bacteroidetes/Firmicutes ratio; (G) composition of the gut microbiota at the phylum level; (H) composition of the gut microbiota at the genus level.

The species accumulation curve is a useful tool for analyzing community composition, species richness, and sampling adequacy. In this study, the end of the slope of the species accumulation curve tended to be flat, indicating that the sampling rate was adequate (Figure 3(A)).

In the PLS-DA, the microbiota structure of the 5/6Nx model group was clearly different from that of the sham- and PSH-treated groups (Figure 3(B)). In the α-diversity analyses, the Shannon and Chao indices were lower in the 5/6Nx model group and significantly higher (*p*<.05) in the Psyllium L than sham group (Figure 3(C,D)). However, the Simpson index was higher in the 5/6Nx model group, and significantly lower (*p*<.05) in the Psyllium L group, than in the sham group (Figure 3(E)).

At the phylum level, Firmicutes and Bacteroidetes were the most abundant species and accounted for the majority of the gut microbiota in the three groups of rats. Compared to the sham group, Firmicutes abundance was significantly reduced, and Bacteroidetes abundance was significantly increased, in the 5/6Nx model group. The ratio of Bacteroidetes/Firmicutes was higher in the 5/6Nx than sham group, and lower in the Psyllium L than 5/6Nx group (Figure 3(F,G)).

**Figure 4.**
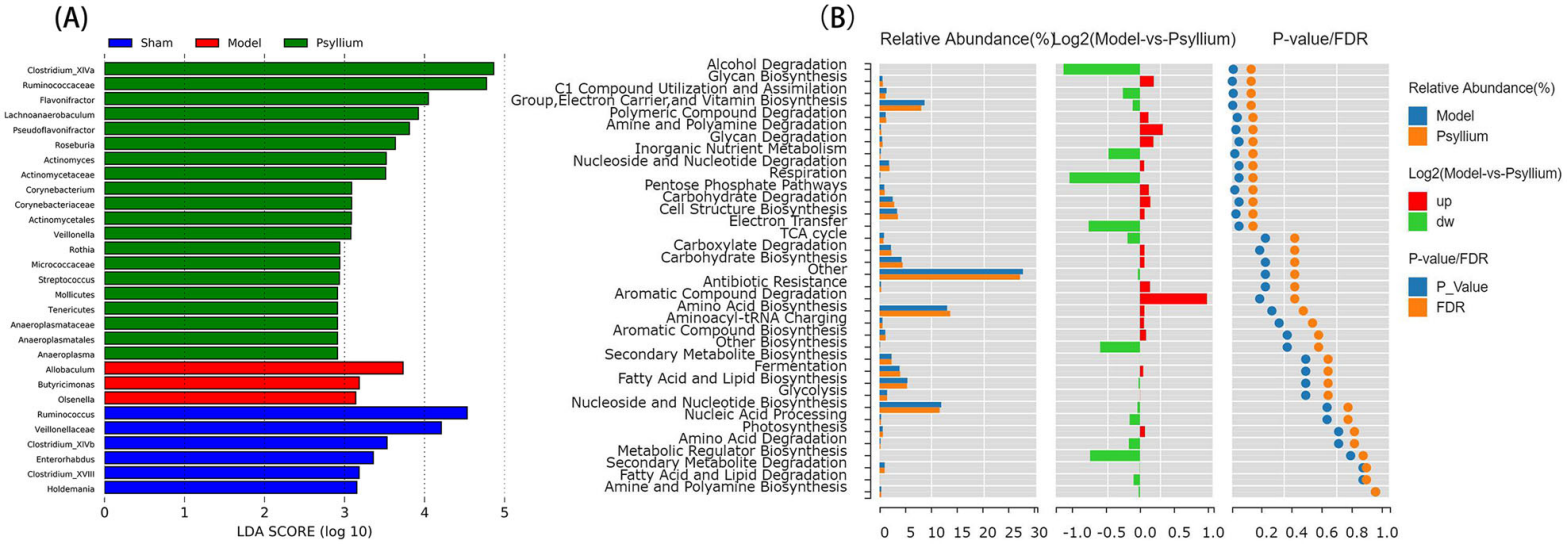
Metabolic analysis based on gut microbiome. (A) Linear discriminant analysis of effect size of bacteria with significant effects on different groups. (B) MetaCyc function analysis of PSH using Wilcoxon’s test.

An LEfSe analysis implied that PSH treatment significantly impacted the gut microbiota of 5/6Nx rats (Figure 4(A)); it increased the relative abundances of *Acetatifactor*, *Turicibacter*, *Ruminococcus*, *Oscillibacter*, and *Saccharibacter*, and decreased the relative abundance of *Methanobrevibacter*, *Romboutsia*, *Blautia*, *Dorea*, and *Bacteroides* (Figure 3(H)).

A MetaCyc function analysis using Wilcoxon’s test showed that polymeric compound degradation, secondary metabolite biosynthesis, amine and polyamine degradation and biosynthesis, aromatic compound biosynthesis, fermentation, and glycan degradation differed significantly between the 5/6Nx and Psyllium L groups (Figure 4(B)).

### 3.5. PSH supplementation improves intestinal barrier function

**Figure 5.**
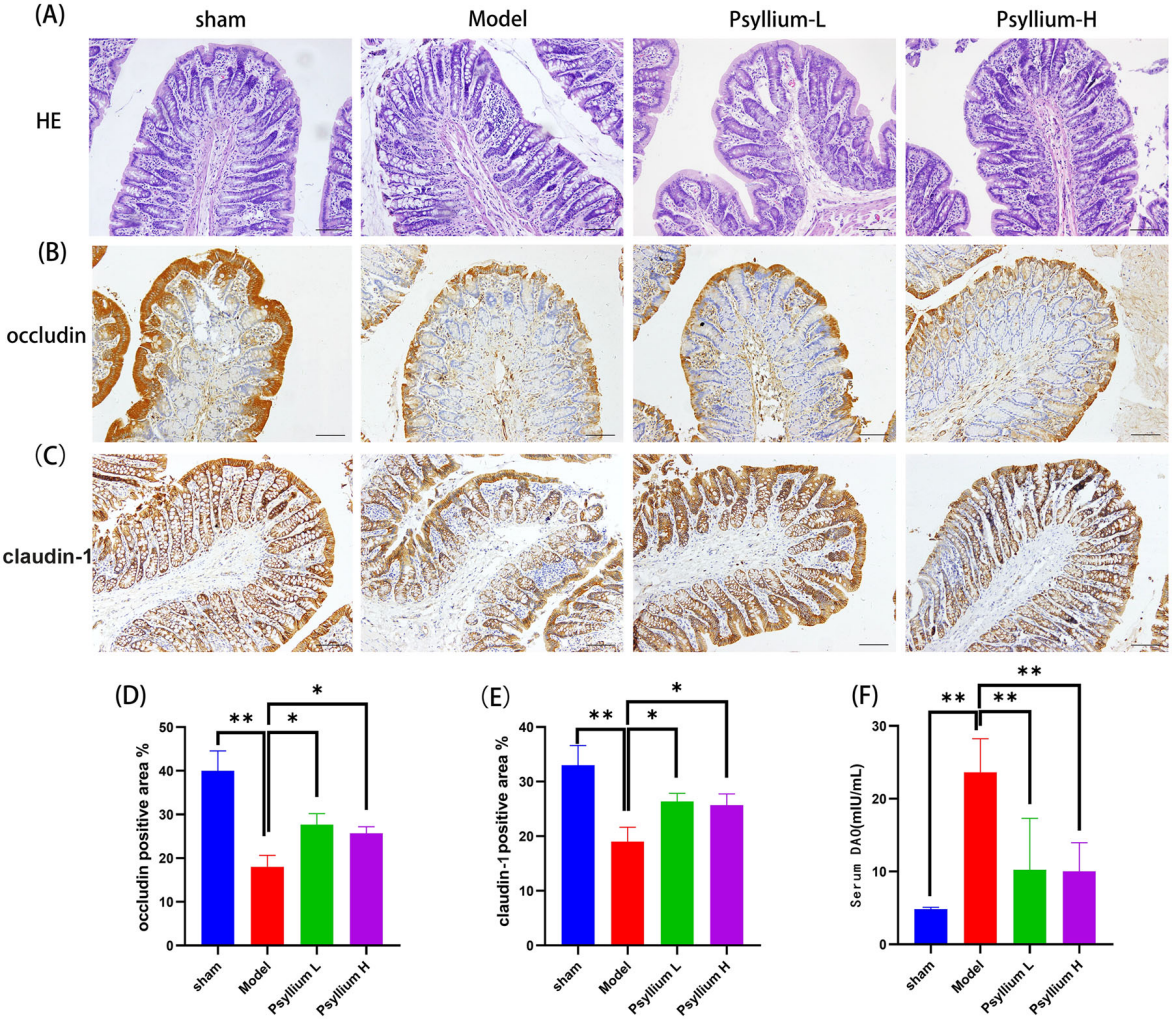
PSH improves intestinal barrier integrity in 5/6Nx rats. (A) H&E staining of colon tissue (×100); (B–E) immunohistochemical analysis of tight junction proteins of the intestinal mucosal barrier (occludin and claudin-1) in colon tissue; (F) serum level of diamine oxidase. Data are presented as mean ± standard deviation (*p<.05, **p<.01).

Mucosal injury and lymphocyte/monocyte infiltration in colon tissue, as visualized by H&E staining, were much higher in 5/6Nx than sham rats. However, in Psyllium L rats, injury of the mucosal layer was significantly ameliorated, and inflammatory infiltration of the mucosal layer was reduced (Figure 5(A)).

The expression of occludin and claudin-1, which are both markers of the colonic mucosal barrier, was significantly lower in 5/6Nx than sham rats *(p*< .01) and significantly higher in Psyllium L and Psyllium H rats than 5/6Nx model rats (*p*< .05; Figure 5(B–E)). Similarly, the serum DAO level was significantly higher in 5/6Nx than sham rats, and significantly lower in PSH-L and PSH-H rats (Figure 5(F)).

## 4. Discussion

Besides its effects on the kidney, CKD can disrupt the gut microbiota, resulting in significant reductions in the number and type of beneficial bacteria, such as *Bifidobacteria* and *Lactobacillus*, and increases in the number and type of potentially pathogenic bacteria, such as *Enterobacteria* and *Enterococcus* [5]. A recent study of the gut microbiota of CKD patients showed higher abundances of bacterial families expressing urease, uricase, indophenol-generating enzyme, and para-cresol-producing enzyme, and reduced abundances of bacteria expressing short-chain fatty acid (SCFA)-producing enzymes [16].

The gut microbiota imbalance associated with CKD promotes the fermentation and degradation of nitrogenous compounds in the intestinal tract, thus generating a variety of potentially toxic metabolites such as ammonia, amines, indoles, phenols, para-cresol sulfate (PCS), and trimethylamine N-oxide. After their absorption into the bloodstream, these uremic products can further exacerbate renal injury and are thus closely associated with the complications of CKD [17,18].

The normal intestinal epithelial barrier consists of a continuous layer of intestinal epithelial cells and tight junctions (TJs); the latter are multi-protein complexes consisting mainly of transmembrane proteins, including claudin-1 and occludin, and cytoplasmic proteins. TJs play an important role in regulating the permeability of the intestinal mucosa by allowing the passage only of ions and small molecules, and not intestinal bacteria or endotoxins [19]. A previous study [20] found significantly lower levels of intestinal claudin-1 and occludin, and increased intestinal barrier permeability, in CKD rats than in healthy rats. Intestinal barrier impairment promotes the translocation of enteric bacteria and endotoxins. The resulting activation of immune cells induces the production of pro-inflammatory cytokines, thus triggering persistent systemic inflammation [17]. The latter not only exacerbates renal fibrosis, but is also strongly associated with cardiovascular and all-cause mortality in CKD [19].

Targeting the gastrointestinal tract to treat kidney disease is not a new strategy but is increasingly gaining acceptance. One clinical method to treat CKD through the gut is intestinal dialysis [21,22]; however, its invasive nature and limited understanding of its consequences have inhibited its broader clinical adoption. However, gut-specific CKD therapy is being reconsidered, as high-throughput technologies have yielded insights into the metabolic potential of the gut microbiota. A number of drug-based therapies have thus been proposed to modify gut microbial metabolism, including α-glucosidase inhibitors [23] and antibiotics [24]. However, diet-based interventions may be a more attractive target due to their generally non-toxic nature.

According to their main nutrient substrates, the gut microbiota can be divided into carbohydrate-fermenting and protein-fermenting bacteria [25]. Sufficient dietary fiber intake promotes the proliferation of carbohydrate-fermenting bacteria and inhibits that of protein-fermenting bacteria. Under these conditions, fiber fermentation results mostly in the production of SCFAs, which provide the energy needed for the growth of intestinal bacteria while reducing the production of toxic metabolites. However, when dietary fiber intake is insufficient, nitrogenous compounds become the main nutrient substrates of the gut microbiota, which favors protein-fermenting bacteria and inhibits carbohydrate-fermenting bacteria. Among the toxic metabolites produced by the former bacteria are IS and PCS [26]. Thus, an adequate supply of dietary fiber prevents protein fermentation, and therefore the production of IS and PCS, by regulating the composition and metabolism of the gut microbiota.

During CKD, urea in the intestinal cavity is metabolized by urea-utilizing bacteria, potentially leading to gut microbiota dysbiosis and intestinal barrier destruction [27]. Dietary fiber fermentation reduces not only intestinal urea, by inhibiting protein-fermenting intestinal bacteria, but also serum urea. The decrease in intestinal urea levels protects TJ proteins in the intestinal tract [9]. Dietary fiber also increases fecal volume, as the reduction in protein-fermenting bacteria causes the excess nitrogen to be released in the feces [28]. The reduction in protein-fermenting bacteria mediated by dietary fiber enhances the barrier function of intestinal mucus by protecting it from degradation [29,30].

PSH is a water-soluble gelatinous mucilage extracted from the plant *Plantago ovata* [11]. It is a highly branched, gel-forming arabinoxylan and polymer rich in arabinose and xylose [13]. Among the nutritional benefits of PSH are reductions in the glycemic index, the risk of cardiovascular disease, cholesterol levels, and constipation, as well as positive effects on the gut microbiota [14, 31]. In our study, PSH supplementation decreased SCr and BUN levels, and ameliorated renal tubular interstitial injury in 5/6Nx rats. The satiating effect of PSH was evidenced by the decrease in food intake/body weight in 5/6Nx rats after PSH supplementation. The 16SrRNA sequencing analysis performed in this study indirectly showed that PSH could prevent gut microbiota disruption, improve intestinal barrier function, and down-regulate serum IS, IL-1β and IL-6 levels.

The arabinose and butyrate produced by PSH degradation may also act as active components [32], as they are further fermented to produce SCFAs [33]. The ability of PSH supplementation to decrease the ratio of Bacteroidetes/Firmicutes in the gut of 5/6Nx rats suggested that PSH alleviates CKD-induced adverse changes in the gut microbiota by acting at the phylum level. PSH increases the relative abundance of *Acetatifactor*, *Turicibacter*, *Ruminococcus*, *Oscillibacter*, and *Saccharibacter* and decreases the relative abundance of *Methanobrevibacter*, *Romboutsia*, *Blautia*, and *Dorea. Turicibacter* is involved in fermentation [34], *Oscillibacter* produces SCFAs [35], and *Saccharibacter* may reduce SCr levels and increase estimated glomerular filtration rates [36]. Meanwhile, *Dorea* is a common pathogen [37]. A MetaCyc functional analysis showed that polymeric compound degradation, secondary metabolite biosynthesis, amine and polyamine degradation and biosynthesis, aromatic compound biosynthesis, fermentation, and glycan degradation differed significantly between the 5/6Nx and PSH rat groups in this study. These results suggest that PSH has positive effects on polymeric compound degradation, amine and polyamine degradation and biosynthesis, aromatic compound biosynthesis, fermentation, and glycan degradation. As shown in Figure 3(C–E), Shannon’s and Chao’s indices were higher, and Simpson’s index was lower, in PSH than sham and model rats. Shannon’s index primarily assesses the abundance of dominant species, while the Simpson assesses the abundance of rare species. Thus, PSH seems to act by increasing the abundance of dominant species.

Intestinal barrier function depends on the mucosal integrity maintained by TJs between intestinal epithelial cells [38]. A disturbance of the expression of TJ proteins, including occludin and claudin-1, indicates damage to the intestinal mucosa and thus potentially an increase in the permeability of the intestinal epithelium, which would allow bacterial translocation and an increase in the serum DAO level [39]. DAO is an intracellular enzyme in the intestinal epithelium [40] that usually has a low blood level. Disruption of the intestinal mucosal barrier, which results in an increase in intestinal permeability, allows the release of DAO into the lumen and, eventually, the peripheral blood [41]. Our study showed that orally administrated PSH improves intestinal barrier function by modulating mucosal structures and up-regulating the expression of TJ proteins, thereby reducing the level of DAO released from the colon into the bloodstream.

In summary, this study demonstrated that host–diet–microbiota interactions can delay CKD progression. Specifically, it showed that PSH supplementation can improve intestinal microecology while also reducing both the synthesis of uremic compounds and systemic inflammation.

## 5. Conclusions

This study presents new and clinically relevant concepts linking dietary fiber and gut microbiota dysbiosis to CKD. By targeting the gut–kidney axis, PSH supplementation may ultimately lead to a paradigm shift in the treatment of CKD that includes a role for dietary fiber, such as PSH.

## Data Availability

The datasets used and/or analyzed in this study are available from the corresponding author upon reasonable request.

## Geolocation information

Location: Guangzhou, Guangdong, China (112.57°E-114.3°E, 22.26°N-23.56°N).
